# *In vivo Nupr1* regulates oxidative stress–endoplasmic reticulum stress crosstalk and mitochondria-dependent apoptosis in varicocele-related testicular injury

**DOI:** 10.1371/journal.pone.0359950

**Published:** 2026-11-04

**Authors:** Xue Jiang, Lemin Tang, Zuzhi Ma, Xinping Wu, Ziyi Zhang, Wenwei Nian, Tianxiang Li, Yaping Liao

**Affiliations:** 1 School of Life Sciences, Bengbu Medical University, Bengbu, China; 2 Anhui Engineering Research Center for Neural Regeneration Technology and Medical New Materials, Bengbu Medical University, Bengbu, China; 3 School of Clinical Medicine, Bengbu Medical University, Bengbu, China; The Islamic University, IRAQ

## Abstract

Varicocele (VCL) is a major cause of male infertility and is closely associated with oxidative stress (OS)-induced testicular injury. Increasing evidence suggests that endoplasmic reticulum stress (ERS) interacts with OS and mitochondrial dysfunction in the pathogenesis of spermatogenic impairment, but the molecular regulators linking these processes remain incompletely understood. In this study, we investigated the role of *Nupr1* in VCL-related testicular stress responses. Re-analysis of transcriptomic data from VCL rat testes identified *Nupr1* as a candidate gene associated with OS, ERS, and apoptosis. *Nupr1* expression was significantly increased in VCL testes and was similarly upregulated in GC-2 and TM4 cells under H_2_O_2_-induced OS or tunicamycin-induced ERS. Stable knockdown of *Nupr1* in GC-2 cells reduced viability under oxidative challenge, increased intracellular ROS accumulation, decreased mitochondrial membrane potential, and enhanced apoptosis, accompanied by increased cleaved caspase-3 expression and an elevated Bax/Bcl-2 ratio. Notably, *Nupr1* deficiency attenuated the adaptive ERS response, as shown by reduced expression of GRP78, IRE1/p-IRE1, PERK/p-PERK, and ATF6. Pharmacological induction of ERS with tunicamycin further worsened cell injury in *Nupr1*-deficient cells, whereas 4-PBA failed to restore cell viability or GRP78 expression. Collectively, these findings indicate that *Nupr1* is involved in the cellular stress response relevant to VCL and may help maintain adaptive UPR signaling and mitochondrial homeostasis in spermatogenic cells under oxidative stress.

## Introduction

Varicocele (VCL) is a common disorder of the male reproductive system and is strongly associated with impaired male fertility. It is widely regarded as one of the major causes of male infertility [1,2]. Among the mechanisms implicated in VCL-associated reproductive dysfunction, oxidative stress (OS) has been consistently recognized as a central pathogenic factor [3–5]. Excessive reactive oxygen species (ROS), generated under conditions such as heat stress, hypoxia/ischemia, venous stasis, and metabolite reflux, can disrupt the testicular microenvironment and impair spermatogenic function [6–8]. Increasing evidence indicates that OS does not act in isolation. Excess ROS can interfere with oxidative protein folding and calcium homeostasis in the endoplasmic reticulum (ER), thereby triggering endoplasmic reticulum stress (ERS) and activation of the unfolded protein response (UPR). In turn, persistent or maladaptive ERS may further enhance ROS production and compromise mitochondrial function, forming an interconnected stress network involving OS, ERS, and mitochondria [9–14]. Evidence from both human studies and animal models of VCL increasingly demonstrates activation of ERS/UPR markers in affected testes and links these pathways to testicular injury and dysfunction [15–17].

Our previous work showed that ERS-related proteins were upregulated in the testes of rats with VCL, and transcriptomic sequencing identified Nuclear protein 1 (*Nupr1*) as a markedly upregulated gene in VCL testes [17]. *Nupr1* is a small stress-inducible intrinsically disordered protein that is responsive to multiple forms of cellular stress, including OS and ERS [18–20]. Previous studies in non-reproductive systems have suggested that *Nupr1* participates in antioxidant defense, metabolic adaptation, and stress-associated transcriptional regulation [18,21,22]. However, its role in the male reproductive system, particularly in VCL-related testicular injury, remains poorly understood.

Recent evidence further suggests that *Nupr1* may be important for male germ cell homeostasis. In mice, loss of *Nupr1* has been associated with age-dependent testicular degeneration, whereas in human testicular pathology, reduced *Nupr1* expression has been linked to germ cell vulnerability [23,24]. These findings raise the possibility that *Nupr1* may function as a stress-adaptive factor in spermatogenic cells. Based on these observations, the present study aims to investigate the potential role of *Nupr1* in the pathophysiological changes of VCL testes, with a particular focus on its relationship with OS, ERS, and mitochondrial dysfunction. By combining transcriptomic re-analysis, *in vivo* validation, and *in vitro* functional experiments, we sought to determine whether *Nupr1* participates in the stress response of testicular cells and to clarify its potential contribution to spermatogenic cell survival under oxidative challenge.

## Materials and methods

### RNA sequencing and bioinformatic analysis

The transcriptome data (GSE344502) from our previous VCL model were re-analyzed to identify differentially expressed genes (DEGs) associated with OS, ERS, and apoptosis [25]. All animal procedures in the previous study were approved by the Ethics Committee of Bengbu Medical University. Venn diagrams were generated to visualize the overlap of these DEGs, and based on literature analysis, target genes were selected. Additionally, WGCNA (Weighted Gene Co-expression Network Analysis) was performed using the R package to assess gene co-expression relationships in the transcriptome data. The Pearson correlation coefficients between genes in the most disease-associated module were calculated, and genes highly correlated with the target gene were selected for subsequent enrichment analysis. The target gene was subsequently validated using preserved testicular tissues from our previous VCL rat model and mouse spermatogenic GC-2 cells as well as testicular Sertoli cell line TM4 *in vitro*.

### Cell culture and construction of lentivirus transfected GC-2 cell line

The GC-2 (spermatogenic) and TM4 (Sertoli) cell lines were obtained from Procell Life Science & Technology (Wuhan, China). Cells were maintained in Dulbecco’s Modified Eagle Medium (DMEM; Procell, Wuhan, China) supplemented with 10% fetal bovine serum (FBS; Procell, Wuhan, China) and 1% penicillin–streptomycin at 37 °C in a humidified atmosphere of 5% CO_2_.

Short hairpin RNA (shRNA) constructs targeting *Nupr1* were designed and packaged by GeneChem (Shanghai, China) in a puromycin-selectable lentiviral backbone (LV-*Nupr1*). Two *Nupr1*-targeting shRNAs were used:sh*Nupr1*–1: target sequence 5′-GAGAGAAGCTGCTGCCAATAC-3′; sh*Nupr1*–2: target sequence 5′-GATCAGTACAGCCTGGCCCAT-3′. A non-targeting shRNA (shNC) served as the negative control. GC-2 cells were maintained in complete growth medium under standard conditions (37 °C, 5% CO_2_). For transduction, cells were seeded into 6-well plates at 2 × 10^5^ cells/well and allowed to adhere overnight. The following day, culture medium was replaced with fresh medium containing polybrene (5 µg/mL) and gradient-diluted lentivirus. After 48 h of exposure, cells were switched to selection medium supplemented with puromycin to eliminate non-transduced cells. Selection medium was refreshed after 24–48 h, followed by maintenance in complete medium. The optimal multiplicity of infection (MOI) was determined empirically by monitoring transduction efficiency via fluorescence microscopy. Using the selected MOI, transductions were repeated, and successfully infected cells were expanded for downstream experiments. Knockdown efficiency (mRNA and protein) was verified by RT-qPCR and Western blot in subsequent assays (see corresponding sections).

### Cell treatment

To induce OS, GC-2 cells were treated with hydrogen peroxide (H_2_O_2_; 30% stock, analytical grade) for 12 hours. Immediately before each experiment, H_2_O_2_ was freshly diluted in complete culture medium to the specified concentrations. To investigate the interplay between ERS, OS, and *Nupr1* knockdown, we utilized two ERS modulators: 4-phenylbutyric acid (4-PBA; Solarbio, Beijing, China), a chemical chaperone serving as an ERS inhibitor, and tunicamycin (TM; Cell Signaling Technology, USA), an ERS inducer. Both compounds were prepared according to the manufacturers’ instructions and diluted to their respective working concentrations in culture medium (1 mM for 4-PBA and 2 ng/mL for TM).

Furthermore, to evaluate the effect of *Nupr1* knockdown on cell viability under OS and to explore the associated cell death pathways, GC-2 cells were pretreated with the pan-caspase inhibitor Z-VAD-FMK (APExBIO Technology LLC, USA) or the necroptosis inhibitor necrosulfonamide (MedChemexpress LLC, USA). The inhibitors were applied 30–60 minutes prior to H_2_O_2_ exposure and maintained throughout the treatment at final concentrations of 10 μM and 40 μM, respectively.

### Cell viability and ROS generation assays

The cytotoxicity of H_2_O_2_ toward GC-2 cells was evaluated using the Cell Counting Kit-8 (CCK-8; Biosharp, Beijing, China) according to the manufacturer's protocol. Briefly, cells were seeded in 96-well flat-bottomed plates at a density of 5 × 10^3^ cells per well and subjected to the designated treatments for the specified durations. Following treatment, the cells were incubated with CCK-8 solution for 2 h. Absorbance at 450 nm was subsequently measured using a microplate reader (BioTek, USA) to determine cell viability.

Intracellular ROS levels were measured using a Fluorometric Assay Kit (Elabscience, Wuhan, China). GC-2 cells were cultured in six-well plates for 48 h, then harvested by digestion and centrifugation. The cell pellet was resuspended in a freshly prepared solution of Reagent 2 (Dihydroethidium, DHE) and incubated at 37 °C for 40 min. After incubation, unbound probe was removed by washing with Reagent 1 (buffer). Fluorescence was quantified by flow cytometry within 1 h.

### Detection of apoptosis

Apoptosis was assessed using two complementary methods. First, GC-2 cell apoptosis was evaluated with an Annexin V-APC/PI Apoptosis Detection Kit (KeyGEN BioTECH, Nanjing, China) according to the manufacturer's protocol. Briefly, cells from three experimental groups (shNC-H_2_O_2_, sh*Nupr1–1*-H_2_O_2_, and sh*Nupr1–2*-H_2_O_2_) were collected, stained with Annexin V-APC and propidium iodide in the dark for 10 min at room temperature, and then analyzed within 20 min on a Cytomics FC500 flow cytometer (Beckman Coulter, USA). A minimum of 10,000 events per sample were acquired, and data were processed using the accompanying CXP analysis software (Beckman Coulter).

Second, apoptosis was visually confirmed via the (Terminal deoxynucleotidyl transferase dUTP nick-end labeling, TUNEL) assay using a one-step kit (Beyotime, Shanghai, China). Briefly, After the indicated treatments, samples were fixed, permeabilized, and incubated with TUNEL reaction solution at 37 °C for 60 min in the dark. Following PBS washes, nuclei were counterstained as appropriate, and fluorescence images were acquired using a fluorescence microscope.

### Fluorescent staining of mitochondria

Mitochondrial membrane potential (ΔΨm) was indirectly assessed by fluorescence staining using the Mito-Tracker Deep Red FM kit (Beyotime). When the GC-2 cells in 6- well plates reached approximately 30% density, the Mito-Tracker Deep Red FM working solutionwas added and incubated at 37°C for 30 min. This working solution was then removed and replaced with a Dulbecco’s modified eagle medium containing 10% FBS. The cells were observed using an inverted fluorescence microscope (Mshot, Guangzhou, China). The captured images were then processed using the ImageJ software (National Institutes of Health, Bethesda, MD, USA)

### ER Immunofluorescence detection

GC-2 cells were seeded in 6-well plates at a density of 2 × 10^5^ cells per well and subjected to the designated treatments. Following removal of the culture medium, the cells were washed with 500 μL of PBS per well, then incubated with 1 mL of ER Tracker Red (Beyotime) working solution at 37 °C for 20 min. Subsequently, the nuclei were counterstained with 4′,6-diamidino-2-phenylindole (DAPI; Biosharp, Beijing, China). Fluorescence images were acquired using an inverted fluorescence microscope and processed with ImageJ software.

### RNA isolation, RT-qPCR, protein extraction and western blotting

Total RNA was extracted from testicular tissue using TRIzol reagent (Ambion, USA). After removal of genomic DNA, mRNA was reverse transcribed into cDNA using the SweScript All-in-One RT SuperMix for qPCR kit (Servicebio, Changsha, China). Quantitative real-time PCR (RT-qPCR) was performed on an ABI system using the SYBR Green qPCR kit (Servicebio). The relative expression level of *Nupr1* mRNA was normalized to the endogenous control *β-actin*. Primer sequences were: *Nupr1* (F: 5′-GAGACGGAGCTGGAGATAAGG-3′, R: 5′-GTGGGAAAGAATCAA-3′); *β-actin* (F: 5′-TTCTACAATGAGCTGCGTGTG-3′, R: 5′-GGGGTGTTGAAGGTCTCAA-3′). The thermal cycling conditions were 95°C for 5 min, followed by 40 cycles of 95°C for 15 s and 60°C for 30 s. The fold-change for each mRNA was calculated by using the 2^–ΔΔCt^ method.

Protein extracts from GC-2 cells, TM4 cells, and rat testes were prepared using ice-cold RIPA lysis buffer (Beyotime) supplemented with protease inhibitor cocktail (Biosharp). The lysates were centrifuged at 12,000 rpm and 4 °C for 10 min, and the protein concentration in the resultant supernatant was determined with a bicinchoninic acid (BCA) assay kit (Beyotime). Proteins (20 µg per lane) were separated by SDS-PAGE on 10% gels (or 15% gels for low-molecular-weight proteins) at 120 V for 100 min and then transferred onto nitrocellulose membranes (Biosharp).

After blocking with 5% non-fat milk or 5% BSA (for phospho-specific antibodies) for 2 h at room temperature, the membranes were incubated overnight at 4 °C with the following primary antibodies: NUPR1 (1:300, Proteintech), GRP78 (Glucose regulatory protein 78, 1:1000, ABclonal), IRE1 (Inositol requiring enzyme 1, 1:1000, ABclonal), p-IRE1 (1:800, ABclonal), PERK (Protein kinase R-like endoplasmic reticulum kinase, 1:1000, ABclonal), p-PERK (1:1000, ABclonal), Chop (C/EBP Homologous Protein, 1:600, ABclonal), cleaved-caspase-3 (1:300, Servicebio), caspase-12 (1:800, ABclonal), Bcl2 (B-cell lymphoma 2, 1:1000, ABclonal), Bax (Bcl-2 associated X protein, 1:1000, ABclonal), ATF6 (Activating transcription factor 6, 1:1000, ABclonal), and β-actin (1:10000, ABclonal). Following three washes with TBST, the membranes were incubated with a horseradish peroxidase (HRP)-conjugated anti-rabbit secondary antibody (1:10000, ABclonal) for 2 h at room temperature with gentle shaking.

Protein bands were visualized using an ECL substrate and imaged with a Bio-Rad system (Tenon, Shanghai, China). Band intensities were quantified using ImageJ software, with β-actin serving as the loading control. The relative expression of each protein is presented as the fold change compared to the control group.

### Statistical analyses

Data are presented as the mean ± SEM from at least three independent experiments. Statistical analyses were performed using GraphPad Prism 8 software. For comparisons between two groups, an unpaired Student's t-test was used. For comparisons among three or more groups, one-way ANOVA was applied, followed by Tukey's or Dunnett's post hoc test. A *p*-value of less than 0.05 was considered statistically significant.

## Results

### *Nupr1* participates in the regulation of OS and ERS in testes of VCL rats

To identify key genes associated with OS, ERS, and apoptosis in VCL, we re-analyzed our previously generated testicular transcriptomic dataset from a rat VCL model. DEGs related to these biological processes were screened, and their overlap was visualized using Venn diagrams. Based on this integrative analysis together with literature review, *Nupr1* was identified as a candidate target gene (Fig 1A). Weighted gene co-expression network analysis (WGCNA) identified six co-expression modules. Among them, the turquoise module showed the strongest association with VCL and was therefore selected for further analysis (Fig 1B). Pearson correlation analysis was then performed within this module, and genes with a correlation coefficient greater than 0.95 with *Nupr1* were subjected to enrichment analysis. These highly correlated genes were significantly enriched in apoptotic signaling pathways (Fig 1C), suggesting that *Nupr1* may be involved in VCL-associated testicular spermatogenic cell injury through apoptosis-related mechanisms.

**Fig 1:**
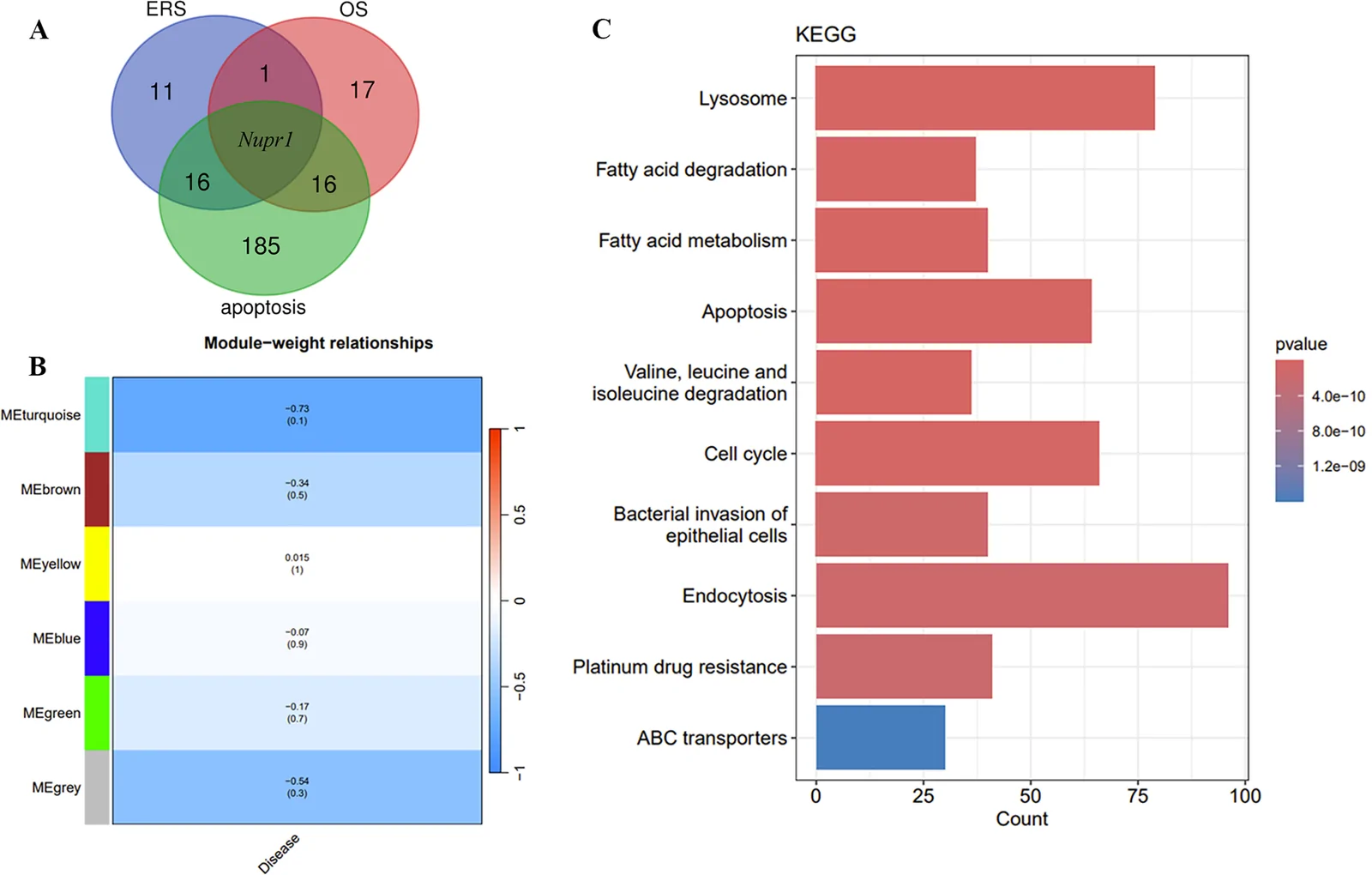
Bioinformatic mining identifies *Nupr1* as a key regulator linked to apoptosis in VCL. (A) *Nupr1* was identified as a common differentially expressed gene through Venn diagram analysis; (B) The WGCNA network demonstrated a disease-associated module containing *Nupr1*; (C) Enrichment analysis revealed that genes highly correlated with *Nupr1* were significantly enriched in apoptotic signaling pathways.

To validate this finding *in vivo*, *Nupr1* expression was examined in testicular tissues from VCL rats. Both mRNA and protein levels of *Nupr1* were significantly increased compared with those in control testes (Fig 2A), indicating that *Nupr1* is induced under VCL-related pathological conditions. To further assess whether *Nupr1* responds to OS and ERS *in vitro*, GC-2 and TM4 cells were exposed to H_2_O_2_ or tunicamycin, respectively. In both cell lines, H_2_O_2_ treatment induced a significant increase in *Nupr1* protein expression (Fig 2B). Similarly, tunicamycin treatment significantly upregulated *Nupr1* expression in both GC-2 and TM4 cells (Fig 2C). Together, these results indicate that *Nupr1* is responsive to both oxidative and ERS and may participate in the cellular stress response associated with VCL.

**Fig 2:**
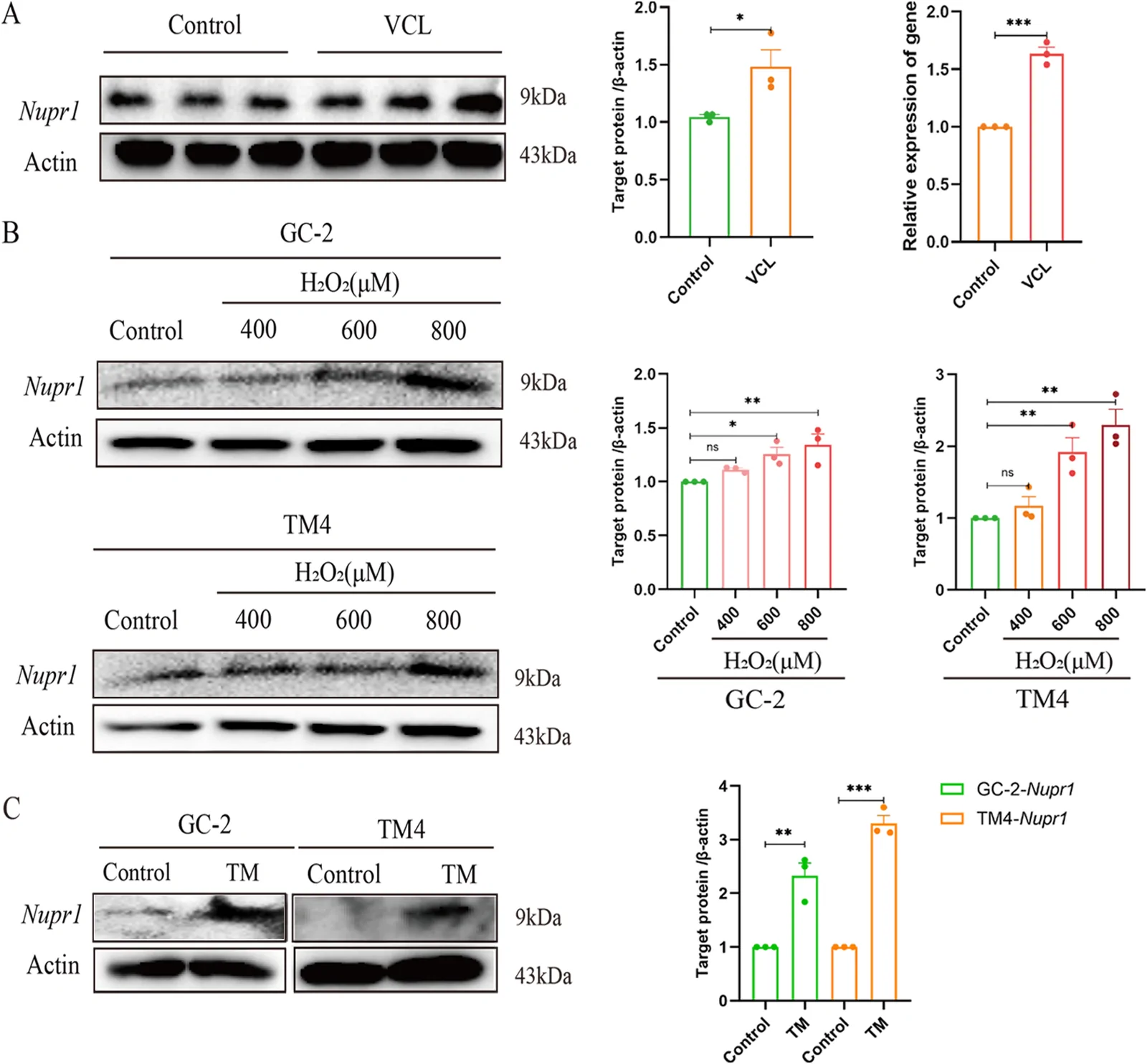
Validation of differential *Nupr1* expression in testicular tissues and two testis cell lines. (A) *Nupr1* expression at both the mRNA and protein levels in testicular tissues from the VCL model; (B) *Nupr1* expression in GC-2 and TM4 cell lines under H_2_O_2_-induced oxidative stress; (C) *Nupr1* expression in GC-2 and TM4 cell lines under tunicamycin (TM)-induced ERS; Data are presented as mean ± SEM. ns, not significant; *p < 0.05, ** *p* < 0.01, *** *p* < 0.001 compared to the control group.

### *Nupr1* knockdown promotes mitochondria-associated apoptosis in GC-2 cells under OS

To examine the functional role of *Nupr1* in spermatogenic cells, stable GC-2 cell lines with *Nupr1* knockdown were established using lentiviral shRNA. A negative control line (GC-2/shNC) and two independent knockdown lines (GC-2/sh*Nupr1–1* and GC-2/sh*Nupr1–2*) were generated. RT-qPCR and Western blot analyses confirmed that both sh*Nupr1* constructs significantly reduced *Nupr1* mRNA and protein levels compared with the shNC control (both *p* < 0.05), with highly consistent knockdown efficiency between the two shRNAs, indicating successful establishment of stable *Nupr1*-deficient GC-2 models (Fig 3A, 3B).

**Fig 3:**
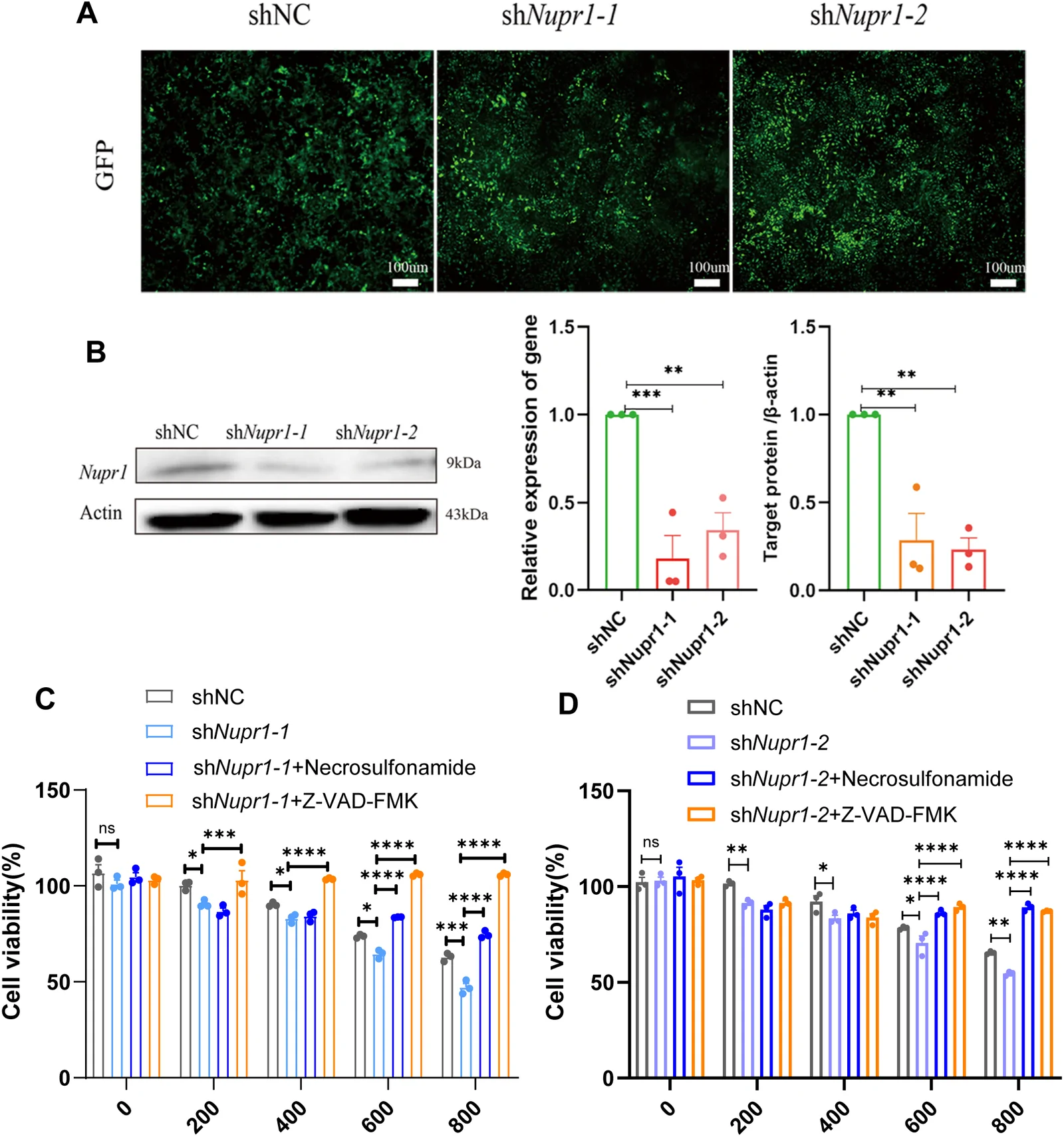
Effect of *Nupr1* knockdown on the viability of H_2_O_2_-treated GC-2 cells. (A) Representative fluorescent images showing GFP expression to confirm lentiviral transduction; (B) The knockdown efficiency of *Nupr1* was validated by RT-qPCR and Western blot analysis. Both sh*Nupr1-1* and sh*Nupr1-2* constructs successfully knocked down *Nupr1* expression compared to the shNC control group. Data are presented as mean ± SEM. * *p* < 0.05, ** *p* < 0.01, *** *p* < 0.001 versus shNC group; (C, D) Cell viability was assessed by the CCK-8 assay following *Nupr1* knockdown under treatment with H_2_O_2_ at various concentrations. GC-2 cells were pretreated with Z-VAD-FMK or necrosulfonamide to investigate the associated cell death pathways. Data are presented as mean ± SEM. ns, not significant; * *p* < 0.05, ** *p* < 0.01, *** *p* < 0.001, **** *p* < 0.0001 compared to the 0 μM H_2_O_2_ group.

Under H_2_O_2_-induced OS, CCK-8 assays showed that cell viability was significantly lower in *Nupr1*-knockdown GC-2 cells than in control cells, and decreased in a dose-dependent manner with increasing H_2_O_2_ concentrations (Fig 3C, 3D). Pretreatment with the pan-caspase inhibitor Z-VAD-FMK or the necroptosis inhibitor necrosulfonamide partially improved cell viability and reduced cell death (Fig 3C, 3D), indicating that loss of *Nupr1* increases the susceptibility of GC-2 cells to oxidative injury.

To further characterize the underlying cellular changes, intracellular ROS levels, mitochondrial membrane potential, and apoptosis were assessed. *Nupr1* knockdown significantly increased ROS accumulation in H_2_O_2_-treated GC-2 cells (Fig 4A). Mito-Tracker Deep Red FM fluorescence intensity was decreased, suggesting a reduction in ΔΨm (Fig 4B, 4C). TUNEL staining showed an increased number of TUNEL-positive cells (Fig 4D). These findings suggest that *Nupr1* deficiency aggravates oxidative imbalance and mitochondrial dysfunction while promoting apoptotic cell death.

**Fig 4:**
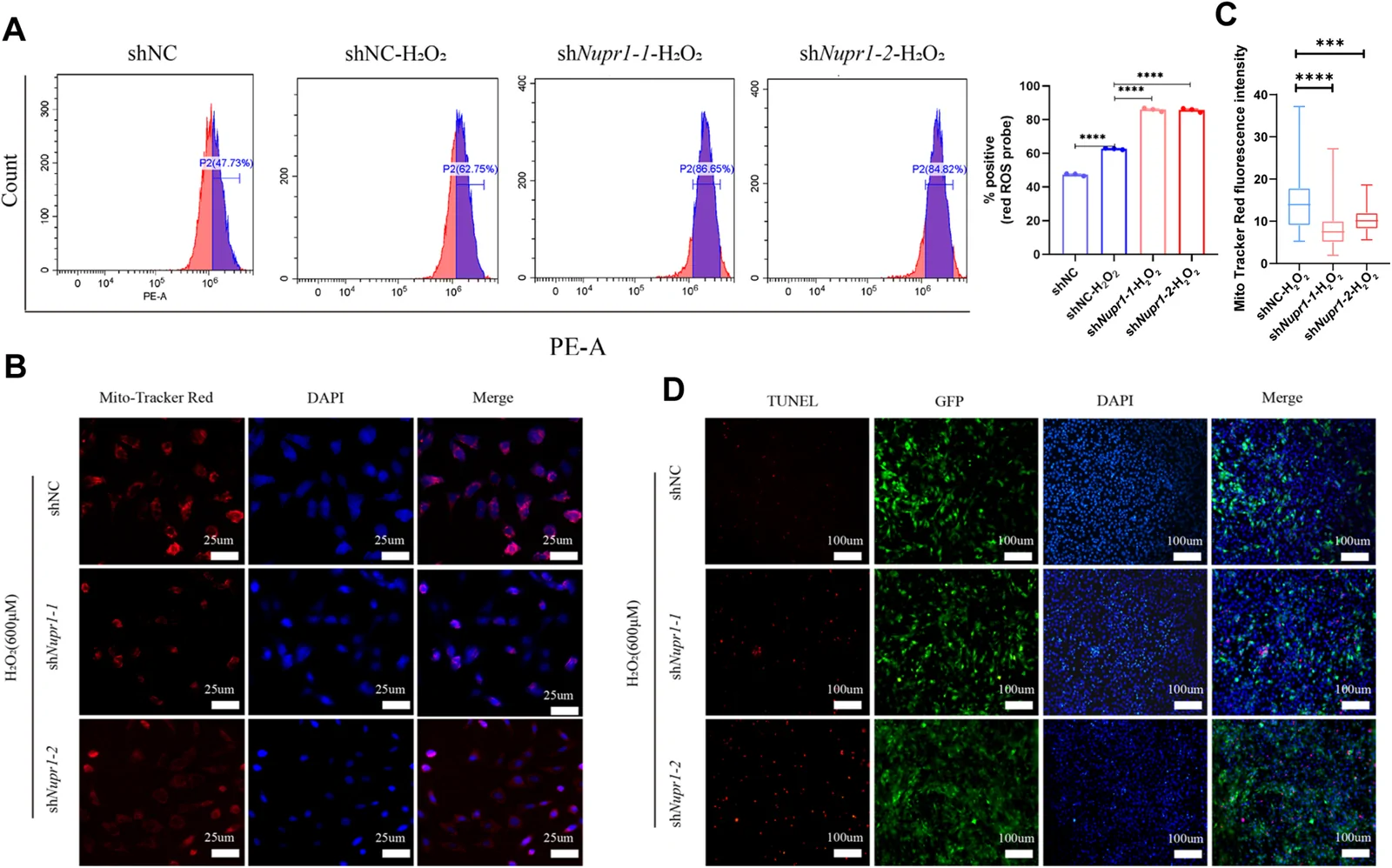
*Nupr1* knockdown increases ROS production and induces mitochondrial damage in H_2_O_2_-treated GC-2 cells. (A) Intracellular ROS levels were measured by flow cytometry using a DHE Redox probe. *Nupr1* knockdown significantly elevated ROS production compared to the shNC control; (B) Representative images of mitochondrial membrane potential-associated fluorescence in GC-2 cells. In *Nupr1*-knockdown cells, reduced red fluorescence was observed, indicating a decrease in mitochondrial membrane potential (ΔΨm); (C) Fluorescence intensity analysis of (B); (D) Apoptosis was detected by TUNEL assay. *Nupr1*-knockdown cells exhibited elevated TUNEL fluorescence. Data are presented as mean ± SEM. ****p < 0.0001 versus shNC group.

We next examined whether apoptosis in this setting was associated with ERS-related or mitochondrial apoptotic signaling. Compared with control cells, *Nupr1*-deficient GC-2 cells showed reduced expression of caspase-12 and CHOP under OS (Fig 5B), indicating that the ER stress–associated pro-apoptotic branch was not activated. In contrast, the mitochondrial apoptosis-related proteins cleaved caspase-3 and Bax/Bcl-2 ratio were significantly increased (Fig 5B). Flow cytometric analysis also confirmed an increased apoptotic rate in *Nupr1*-knockdown cells (Fig 5A). These results indicate that under oxidative challenge, *Nupr1* knockdown is associated predominantly with activation of mitochondria-related apoptotic signaling rather than the ER–caspase-12 axis.

**Fig 5:**
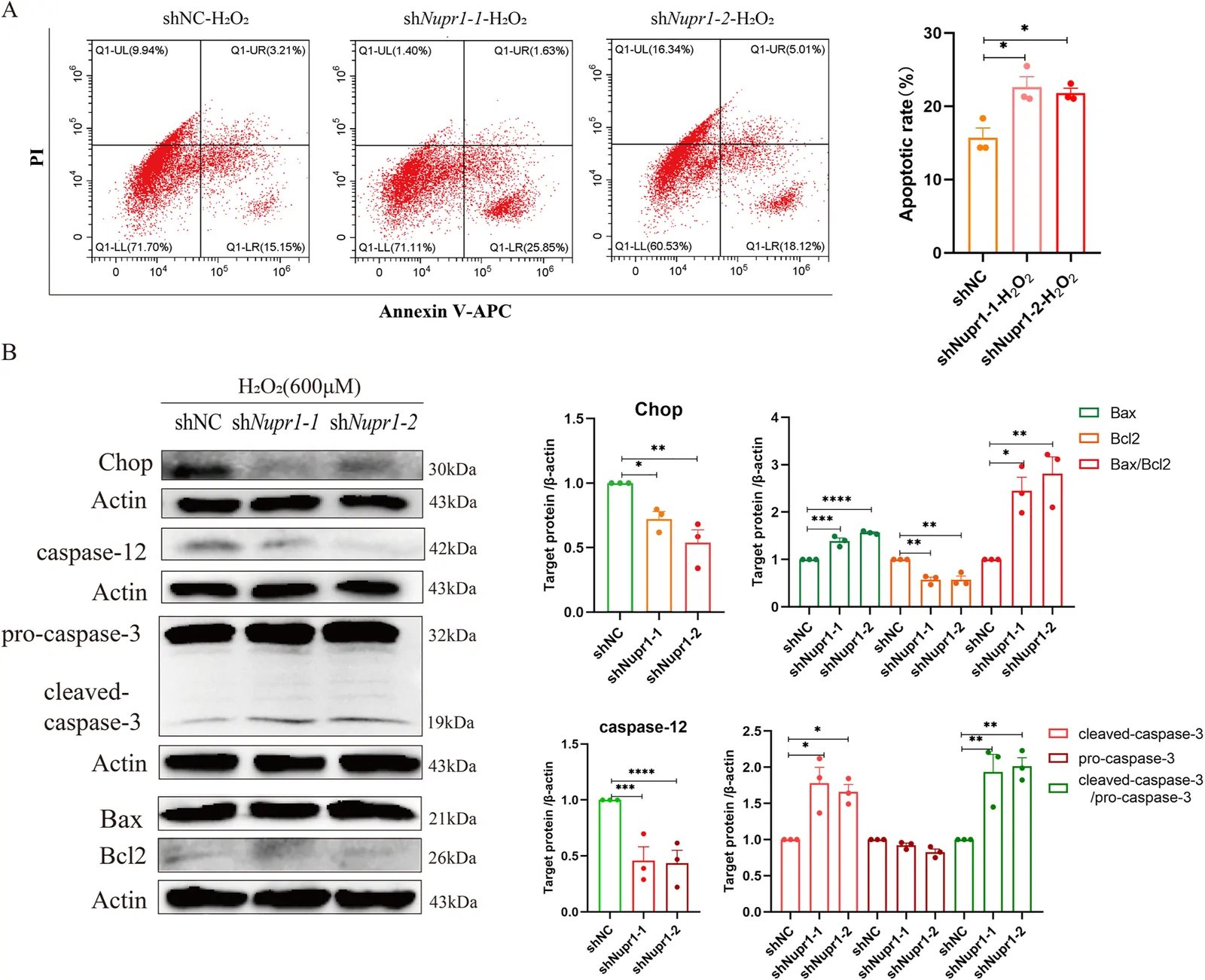
*Nupr1* knockdown triggers mitochondria-associated apoptosis in H_2_O_2_-treated GC-2 cells. (A) Flow cytometric analysis showing an increased apoptosis rate upon *Nupr1* knockdown; (B) Western blot analysis of apoptosis-related proteins, including cleaved caspase-3, Bax, Bcl-2, Chop, and caspase-12, in H_2_O_2_-treated GC-2 cells. Quantitative analysis showed increased cleaved caspase-3 expression and Bax/Bcl-2 ratio, but reduced Chop and caspase-12 expression, in *Nupr1*-knockdown cells. All data are shown as mean ± SEM. * *p* < 0.05, ** *p* < 0.01, *** *p* < 0.001, **** *p* < 0.0001 compared to the shNC group.

### *Nupr1* knockdown attenuates the ERS response in GC-2 cells

To investigate whether *Nupr1* participates in ERS regulation, stable GC-2/shNC and GC-2/sh*Nupr1* cells were exposed to 600 μM H_2_O_2_ for 12 h, and the expression of ERS-related proteins was analyzed by Western blot. Compared with the control group, *Nupr1* knockdown significantly reduced the expression of GRP78, IRE1/p-IRE1, PERK/p-PERK, and ATF6 (Fig 6A, 6B), indicating attenuation of the three major UPR branches in GC-2 cells under OS.

**Fig 6:**
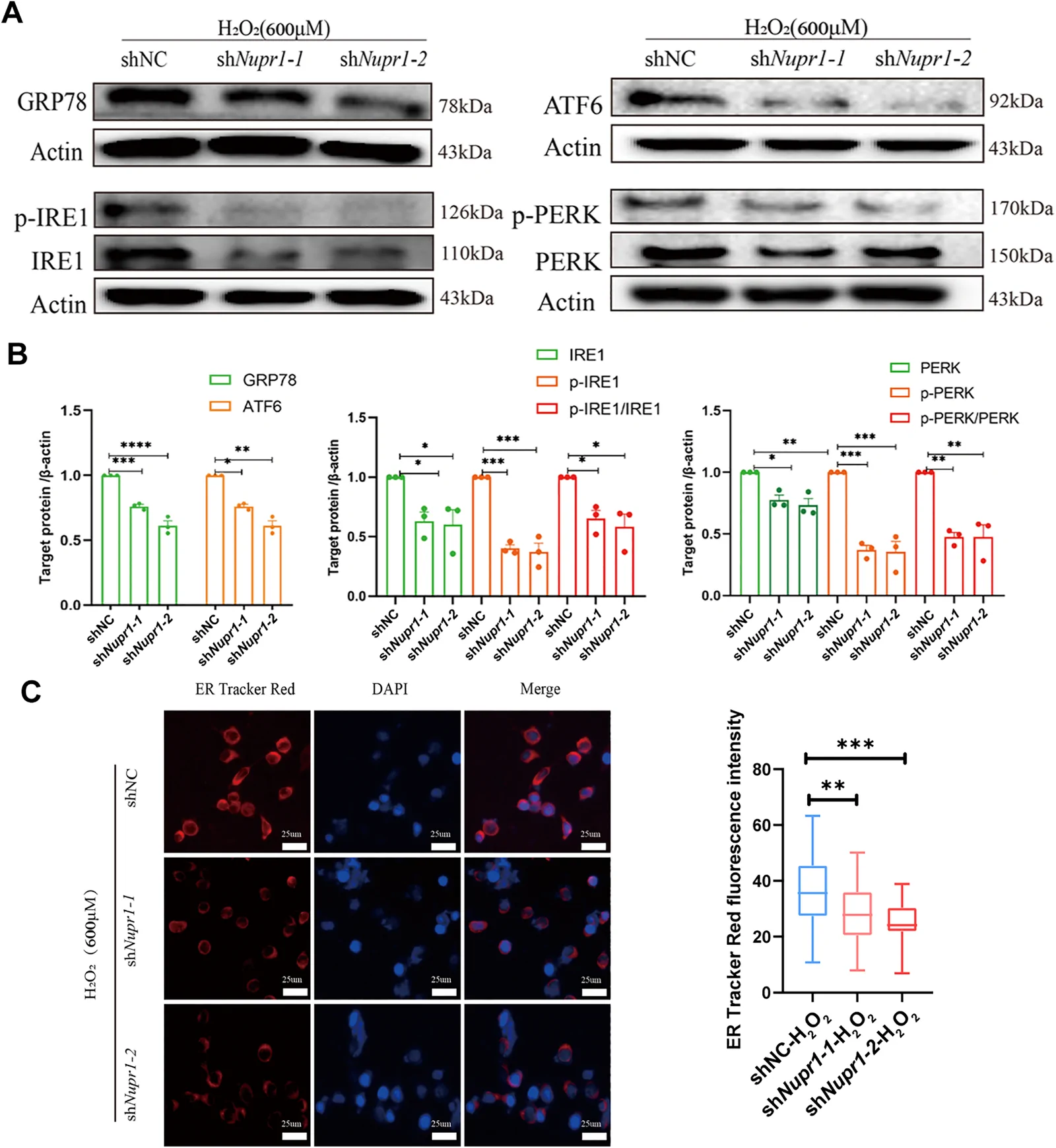
*Nupr1* knockdown attenuates the ERS response in H_2_O_2_-treated GC-2 cells. (A) Representative Western blot images showing protein levels of key ERS markers (GRP78, ATF6, IRE1α, p-IRE1α, PERK, and p-PERK) in *Nupr1*-knockdown cells; (B) Densitometric quantification of the immunoblots from (A); (C) Fluorescent imaging of the ER using an ER-Tracker Red probe, indicating a reduced ERS response upon *Nupr1* knockdown. Nuclei were counterstained with DAPI (blue). Data are presented as mean ± SEM. * *p* < 0.05, ** *p* < 0.01, *** *p* < 0.001, **** *p* < 0.0001 versus the shNC group.

To complement the protein data, ER-tracker fluorescence was used to assess the functional ERS response. Cells transduced with sh*Nupr1–1* or sh*Nupr1–2* displayed substantially weaker red fluorescence than control cells (Fig 6C), consistent with the Western blot findings. Together, these results indicate that *Nupr1* deficiency blunts the adaptive ERS response of GC-2 cells under oxidative challenge.

### Pharmacological modulation of ER stress fails to rescue *Nupr1*-deficient GC-2 cells

To determine whether pharmacological modulation of ERS influences the impaired stress response in *Nupr1*-deficient cells, GC-2/sh*Nupr1* cells were treated with tunicamycin (TM) or 4-phenylbutyrate (4-PBA). TM treatment partially increased GRP78 expression in *Nupr1*-knockdown cells (Fig 7B), suggesting that exogenous ER burden can still stimulate part of the ERS machinery. However, TM treatment further reduced cell viability rather than improving it (Fig 7A), indicating that under conditions of *Nupr1* deficiency, increasing ER protein-folding burden aggravates cellular injury.

**Fig 7:**
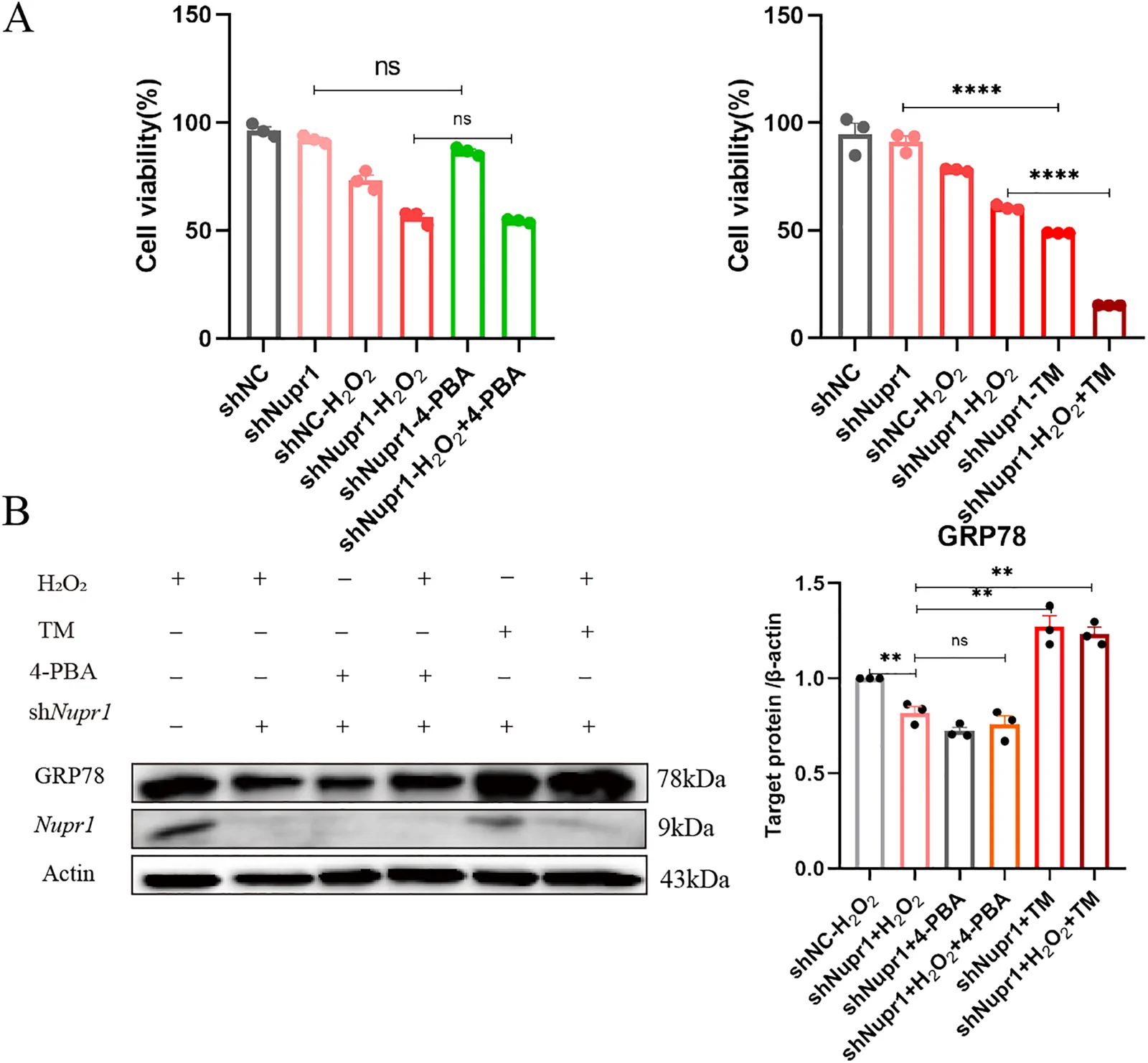
Effects of the ERS modulator 4-PBA and TM on cell viability and ERS in *Nupr1*-knockdown GC-2 cells. (A) Cell viability was measured by the CCK-8 assay following treatment with the chemical chaperone 4-PBA (ERS inhibitor) or tunicamycin (TM, ERS inducer); (B) Western blot analysis of key ERS-related protein GRP78. Data are presented as mean ± SEM. ns, not significant; ** *p* < 0.01, **** *p* < 0.0001 compared to the shNC group.

In contrast, treatment with the chemical chaperone 4-PBA did not restore GRP78 expression or improve cell viability in *Nupr1*-deficient GC-2 cells (Fig 7A, 7B). These findings suggest that in the absence of adequate *Nupr1*-dependent stress adaptation, pharmacological reduction of protein-folding stress alone is insufficient to re-establish an effective ERS response or rescue cell survival.

## Discussion

Varicocele is a major pathological condition associated with male infertility, and OS is widely considered one of the key mechanisms underlying VCL-induced spermatogenic dysfunction. In recent years, increasing attention has been paid to the interplay among OS, ERS, and mitochondrial dysfunction in testicular injury [15,16]. Previous studies in both experimental VCL models and human samples have shown increased ROS production, activation of UPR-related molecules, and enhanced germ cell apoptosis, indicating that these stress pathways are closely interconnected [16,17,26,27]. The present study adds to this framework by showing that *Nupr1* is responsive to both OS and ERS and may participate in stress adaptation in the VCL testis.

Our bioinformatic re-analysis identified *Nupr1* as a candidate gene associated with OS, ERS, and apoptosis in VCL testes. This observation was validated experimentally at both the tissue and cellular levels. Specifically, *Nupr1* was significantly upregulated in VCL rat testes and in GC-2 and TM4 cells exposed to H_2_O_2_ or tunicamycin. These results are consistent with previous reports that *Nupr1* is a stress-inducible protein responsive to various cellular insults [18,19,21,28]. Although *Nupr1* has been studied in cancer biology, metabolic regulation, and general stress adaptation, its role in the male reproductive system remains incompletely understood [18,19,29]. Our findings support the idea that *Nupr1* is involved in testicular stress responses and may be relevant to germ cell adaptation under adverse conditions.

Functionally, under H_2_O_2_-induced OS, *Nupr1* knockdown in GC-2 cells resulted in reduced cell viability, a robust increase in ROS, loss of mitochondrial membrane potential (ΔΨm), increased TUNEL positivity, and upregulation of cleaved caspase-3 and the Bax/Bcl-2 ratio. Both the pan-caspase inhibitor Z-VAD-FMK and the necroptosis inhibitor necrosulfonamide partially alleviated cell death, indicating that *Nupr1*-deficient spermatogenic cells are more susceptible to oxidative injury and may engage multiple cell death programs. It is noteworthy that the effect of *Nupr1* on cell fate appears to be highly context-dependent. In some cell types, such as pancreatic cancer cells or fibroblast-like cells, *Nupr1* deficiency disrupts ER and mitochondrial homeostasis and preferentially induces atypical programmed necrosis or parthanatos rather than classical apoptosis [21,30]. In contrast, in endothelial, neuronal and cardiac-related cells, *Nupr1* has been implicated in canonical mitochondria-dependent, caspase-mediated apoptosis, where its pro-survival or pro-death role varies according to cell type, stress condition, and metabolic context [31–34]. However, based on the present data, mitochondrial apoptosis appears to be the predominant response in *Nupr1*-deficient GC-2 cells exposed to oxidative challenge, although the involvement of programmed necrosis or parthanatos under distinct stimuli or metabolic conditions cannot be excluded.

An important finding of this study is that *Nupr1* knockdown attenuated, rather than enhanced, the canonical ERS response. Reduced expression of GRP78, IRE1/p-IRE1, PERK/p-PERK, and ATF6, together with decreased ER-tracker fluorescence, indicates that *Nupr1* deficiency blunts the adaptive UPR response in GC-2 cells. Moreover, the ERS-associated pro-apoptotic markers CHOP and caspase-12 were also decreased under oxidative stress, suggesting that the ER stress–driven apoptotic branch was not activated. Instead, the concurrent increase in cleaved caspase-3 and the Bax/Bcl-2 ratio indicates that mitochondrial apoptosis was the predominant cell death pathway. This observation is noteworthy because ERS should not be interpreted as uniformly detrimental. The UPR is inherently biphasic: an appropriate adaptive response promotes restoration of protein homeostasis and cell survival, whereas either prolonged hyperactivation or insufficient activation may contribute to cellular injury [35–38]. Our data are more consistent with the latter scenario, suggesting that *Nupr1* may help maintain an adequate adaptive ERS response under OS.

Based on the current findings, *Nupr1* appears to be associated with coordinated stress adaptation involving the ER and mitochondria. When *Nupr1* is reduced, GC-2 cells exhibit weaker UPR signaling, greater ROS accumulation, mitochondrial depolarization, and increased apoptosis. These observations support a model in which *Nupr1* contributes to cellular stress resilience. However, the present study does not definitively establish a direct causal sequence between impaired UPR signaling and mitochondrial apoptosis. It remains possible that *Nupr1* influences several parallel processes, including redox homeostasis, protein-folding adaptation, and mitochondrial stability, which together shape cell fate under oxidative challenge.

Our pharmacological experiments are consistent with this interpretation. Tunicamycin partially increased GRP78 expression in *Nupr1*-deficient cells but further reduced viability, suggesting that when stress adaptation is compromised, additional ER burden aggravates rather than alleviates injury. Conversely, 4-PBA did not restore GRP78 expression or cell viability, indicating that chemical chaperone support alone is insufficient when the overall *Nupr1*-associated adaptive response is impaired. These findings suggest that *Nupr1* may be required for maintaining a functional stress-response framework, although the precise molecular mechanisms remain to be defined.

Previous studies showed that the testes of *Nupr1* knockout mice appeared morphologically normal at 8 months of age, whereas by 10 months these animals developed a Sertoli cell-only phenotype, although the mechanism underlying this delayed degeneration remained undefined [23]. Our findings provide a possible explanation for this observation. Compared with younger animals, the aging testis is likely exposed to a greater cumulative burden of OS and ERS [39–41]. Under such conditions, stress-inducible upregulation of *Nupr1* may function as an adaptive response that helps preserve proteostasis and germ cell survival. In the absence of *Nupr1*, however, this compensatory capacity may be compromised, rendering germ cells more vulnerable to chronic stress-associated injury and progressive depletion.

Notably, recent studies have further identified *Nupr1* as an important suppressor of ferroptosis [24,28,42]. In the male reproductive system, *Nupr1* deficiency has been reported to impair spermatogonial stem cell proliferation and increase susceptibility to ferroptotic injury, supporting a broader role for *Nupr1* in maintaining germ-cell homeostasis [24]. Interestingly, while VCL has been reported to induce ferroptosis in human spermatozoa [43,44], a rat study found that although testicular iron accumulation increased at 2 and 4 months after VCL induction, no molecular evidence of ferroptosis was detected in testicular cells [45]. These findings raise the possibility that *Nupr1*-dependent stress adaptation may differ between germ cells within the testis and mature spermatozoa. Whether the stress-responsive upregulation of *Nupr1* in VCL testes contributes to limiting ferroptotic damage, and whether this mechanism is absent or attenuated in spermatozoa, merits further investigation.

## Conclusion

In summary, our study identifies *Nupr1* as a stress-responsive regulator linking OS and ERS in VCL testis. *Nupr1* was markedly upregulated in VCL testes and in OS/ERS-induced testicular cell models, whereas its knockdown impaired UPR activation, aggravated ROS accumulation, disrupted mitochondrial homeostasis, and promoted mitochondria-dependent apoptosis in GC-2 cells. These findings suggest that *Nupr1* plays a protective role in spermatogenic cells by maintaining adaptive ERS responses and mitochondrial integrity under OS. Future studies should validate these findings *in vivo* and further clarify the upstream regulators and downstream signaling networks of *Nupr1* in testicular stress adaptation.

## Supporting information

S1 File WB original image. (DOCX)
