# Supplementary material for: In vivo Nupr1 regulates oxidative stress–endoplasmic reticulum stress crosstalk and mitochondria-dependent apoptosis in varicocele-related testicular injury
Source: PLoS One. 2026 Nov 4;21(11):e0359950. doi: 10.1371/journal.pone.0359950 (PMC13734504; doi:10.1371/journal.pone.0359950)
Supplement: Supplementary file 1 [file pone.0359950.s001.docx]

**Figure2**

**2A**

**2B**

**2C**

**Figure 3**

**3B**

**Figure 5**

**5B**

**Figure 6**

**6A**

**Figure 7**

**7B**
